# Supplementary material for: The Impact of Candida albicans in the Development, Kinetics, Structure, and Cell Viability of Biofilms on Implant Surfaces—An In Vitro Study with a Validated Multispecies Biofilm Model
Source: Int J Mol Sci. 2024 Mar 14;25(6):3277. doi: 10.3390/ijms25063277 (PMC10970611; doi:10.3390/ijms25063277)
Supplement: Supplementary file 1 [file ijms-25-03277-s001.zip › ijms-2900447-supplementary.pdf]

Supplementary Table S1. Counts (expressed as mean and standard deviation (SD) and 95% confidence intervals (CI) for means) of microbial species (colony-forming units (CFUs)/mL) determined by quantitative polymerase chain reaction (qPCR) in 24, 48, and 72 h biofilms on dental implants in the presence (test) and absence (control) of *Candida albicans* (n=9), using specific primers and probes directed to the 16S rRNA gene. * $p < 0.05$, statistically significant differences when comparing CFU/mL between test and control biofilms at each time interval. Comparisons between groups were performed considering viable cells and total cells.

Species	Incubation time (hours)	Analysis	Condition	Microbial counts (CFU/ml)				% Viability	
				Mean	(SD)	95% CI		Mean	(SD)
						Lower limit	Upper limit		
Streptococcus oralis	24	Total	Control	1.13×10^{7}	6.71×10^{6}	4.04×10^{5}	1.87×10^{7}		
			Test	1.16×10^{7}	4.69×10^{6}	6.52×10^{6}	1.80×10^{7}		
		Viable	Control	5.85×10^{6}	3.76×10^{6}	1.31×10^{6}	1.10×10^{7}	51.78	14.69
			Test	5.76×10^{6}	3.45×10^{6}	1.23×10^{6}	1.08×10^{7}	46.45	14.67
	48	Total	Control	1.22×10^{7}	4.87×10^{6}	5.41×10^{6}	1.82×10^{7}		
			Test	1.65×10^{7}	6.86×10^{6}	7.20×10^{6}	2.59×10^{7}		
		Viable	Control	6.51×10^{6}	2.71×10^{6}	3.51×10^{6}	1.08×10^{7}	56.54	19.16
			Test	8.69×10^{6}	2.63×10^{6}	5.02×10^{6}	1.15×10^{7}	58.79	23.24
	72	Total	Control	9.43×10^{6}	3.43×10^{6}	4.17×10^{6}	1.35×10^{7}		
			Test	1.24×10^{7}	7.25×10^{6}	7.06×10^{6}	2.98×10^{7}		
		Viable	Control	3.95×10^{6}	1.54×10^{6}	2.21×10^{6}	6.26×10^{6}	45.24	18.15
			Test	8.30×10^{6}*	3.80×10^{6}	3.08×10^{6}	1.48×10^{7}	70.92*	25.05
Actinomyces naeslundii	24	Total	Control	7.58×10^{5}	4.95×10^{5}	1.14×10^{5}	1.31×10^{6}		
			Test	1.01×10^{6}	8.02×10^{5}	9.75×10^{4}	2.18×10^{6}		
		Viable	Control	4.20×10^{5}	3.48×10^{5}	2.58×10^{4}	1.03×10^{6}	66.91	30.38
			Test	5.65×10^{5}	3.90×10^{5}	1.01×10^{5}	1.03×10^{6}	68.79	20.82
	48	Total	Control	5.51×10^{5}	3.48×10^{5}	1.10×10^{5}	1.09×10^{6}		
			Test	9.00×10^{5}	7.45×10^{5}	9.02×10^{4}	2.38×10^{6}		
		Viable	Control	3.33×10^{5}	2.13×10^{5}	1.10×10^{5}	6.61×10^{5}	74.22	37.58
			Test	5.72×10^{5}	3.96×10^{5}	9.98×10^{4}	1.31×10^{6}	83.24	38.63
	72	Total	Control	4.43×10^{5}	2.93×10^{5}	7.39×10^{4}	8.09×10^{5}		
			Test	1.21×10^{6}	9.92×10^{5}	4.18×10^{4}	2.65×10^{6}		
		Viable	Control	1.98×10^{5}	1.21×10^{5}	7.39×10^{4}	4.15×10^{5}	57.1	26.76
			Test	4.11×10^{5}	3.05×10^{5}	5.14×10^{4}	9.60×10^{5}	58.47	39.11
Veillonella parvula	24	Total	Control	5.18×10^{7}	4.45×10^{7}	6.70×10^{6}	1.49×10^{8}		
			Test	3.48×10^{7}	2.11×10^{7}	6.62×10^{6}	5.61×10^{7}		
		Viable	Control	3.31×10^{7}	2.14×10^{7}	6.99×10^{6}	6.49×10^{7}	79.09	28.19
			Test	2.31×10^{7}	1.34×10^{7}	4.97×10^{6}	4.38×10^{7}	76.76	30.06
	48	Total	Control	4.58×10^{7}	3.08×10^{7}	6.79×10^{6}	9.32×10^{7}		
			Test	5.77×10^{7}	4.51×10^{7}	8.90×10^{6}	1.31×10^{8}		
		Viable	Control	2.45×10^{7}	1.35×10^{7}	6.68×10^{6}	4.25×10^{7}	67.78	26.03
			Test	3.57×10^{7}	2.79×10^{7}	5.63×10^{6}	8.43×10^{7}	67.2	17.34
	72	Total	Control	3.04×10^{7}	2.26×10^{7}	5.15×10^{6}	6.81×10^{7}		
			Test	2.31×10^{7}	1.30×10^{7}	5.45×10^{6}	4.44×10^{7}		
		Viable	Control	1.23×10^{7}	1.09×10^{7}	3.49×10^{6}	3.73×10^{7}	50.44	25.03
			Test	1.05×10^{7}	5.73×10^{6}	3.41×10^{6}	1.78×10^{7}	51.21	21.46

Species	Incubation time (hours)	Analysis	Condition	Microbial counts (CFU/ml)				% Viability	
				Mean	(SD)	95% CI		Mean	(SD)
						Lower limit	Upper limit		
Fusobacterium nucleatum	24	Total	Control	1.95x106	$1.04x10^{6}$	$3.64x10^{5}$	$3.27x10^{6}$		
			Test	2.05x106	$8.77x10^{5}$	$1.32x10^{6}$	$4.17x10^{6}$		
		Viable	Control	5.96x105	$4.57x10^{5}$	$4.30x10^{4}$	$2.45x10^{5}$	29.12	13.21
			Test	9.39x105	$6.15x10^{5}$	$1.16x10^{6}$	$1.77x10^{6}$	46.1	18.89
	48	Total	Control	$2.85x10^{6}$	$2.08x10^{6}$	$1.70x10^{5}$	$5.09x10^{6}$		
			Test	$1.10x10^{7*}$	$5.45x10^{6}$	$4.19x10^{6}$	$2.02x10^{7}$		
		Viable	Control	$1.52x10^{6}$	$1.24x10^{6}$	$7.17x10^{4}$	$3.64x10^{6}$	47.42	14.9
			Test	$5.15x10^{6*}$	$3.04x10^{6}$	$1.20x10^{6}$	$8.35x10^{6}$	48.25	12.38
	72	Total	Control	$4.15x10^{6}$	$3.45x10^{6}$	$6.15x10^{4}$	$9.60x10^{6}$		
			Test	$1.35x10^{7*}$	$5.86x10^{6}$	$3.64x10^{6}$	$2.12x10^{7}$		
		Viable	Control	$1.31x10^{6}$	$1.07x10^{6}$	$8.92x10^{3}$	$2.63x10^{6}$	27.42	9.711
			Test	$7.53x10^{6*}$	$5.74x10^{6}$	$4.54x10^{5}$	$1.86x10^{7}$	54.36*	26.21
Porphyromonas gingivalis	24	Total	Control	$3.31x10^{4}$	$1.50x10^{4}$	$5.20x10^{3}$	$4.79x10^{4}$		
			Test	$3.83x10^{4}$	$2.18x10^{4}$	$1.13x10^{4}$	$6.20x10^{4}$		
		Viable	Control	$1.43x10^{4}$	$8.24x10^{3}$	$6.25x10^{3}$	$2.56x10^{4}$	40.27	14.91
			Test	$2.21x10^{4}$	$1.27x10^{4}$	$6.41x10^{3}$	$3.87x10^{4}$	57.3	19.82
	48	Total	Control	$2.02x10^{5}$	$1.03x10^{5}$	$7.29x10^{4}$	$3.44x10^{5}$		
			Test	$4.14x10^{5*}$	$1.86x10^{5}$	$1.57x10^{5}$	$6.11x10^{5}$		
		Viable	Control	$6.51x10^{4}$	$3.21x10^{4}$	$2.84x10^{4}$	$1.15x10^{5}$	37.67	17.43
			Test	$2.25x10^{5*}$	$1.13x10^{5}$	$7.34x10^{4}$	$4.07x10^{5}$	54.38*	9.468
	72	Total	Control	$3.65x10^{5}$	$1.97x10^{5}$	$1.10x10^{5}$	$5.81x10^{5}$		
			Test	$1.13x10^{6*}$	$5.51x10^{5}$	$6.98x10^{5}$	$2.44x10^{6}$		
		Viable	Control	$1.24x10^{5}$	$5.57x10^{4}$	$4.93x10^{4}$	$2.05x10^{5}$	37.91	9.136
			Test	$6.78x10^{5*}$	$1.84x10^{5}$	$4.75x10^{5}$	$9.28x10^{5}$	65.29*	18.16
Aggregatibacter actinomycetemcomitans	24	Total	Control	$2.82x10^{6}$	$1.03x10^{6}$	$1.35x10^{6}$	$3.75x10^{6}$		
			Test	$2.72x10^{6}$	$8.14x10^{5}$	$1.68x10^{6}$	$4.07x10^{6}$		
		Viable	Control	$1.10x10^{6}$	$4.24x10^{5}$	$1.27x10^{5}$	$1.52x10^{6}$	44.34	23.92
			Test	$1.49x10^{6}$	$3.53x10^{5}$	$9.54x10^{5}$	$1.99x10^{6}$	56.01	7.191
	48	Total	Control	$4.04x10^{6}$	$1.44x10^{6}$	$2.15x10^{6}$	$6.34x10^{6}$		
			Test	$3.79x10^{6}$	$1.24x10^{6}$	$1.45x10^{6}$	$5.41x10^{6}$		
		Viable	Control	$1.38x10^{6}$	$2.43x10^{5}$	$9.64x10^{5}$	$1.76x10^{6}$	38.36	15.88
			Test	$2.11x10^{6*}$	$7.45x10^{5}$	$5.47x10^{5}$	$3.06x10^{6}$	55.28*	10.77
	72	Total	Control	$5.01x10^{6}$	$1.37x10^{6}$	$3.47x10^{6}$	$7.29x10^{6}$		
			Test	$4.90x10^{6}$	$1.65x10^{6}$	$2.52x10^{6}$	$6.60x10^{6}$		
		Viable	Control	$1.56x10^{6}$	$3.28x10^{5}$	$1.21x10^{6}$	$2.16x10^{6}$	32.17	6.379
			Test	$3.15x10^{6*}$	$3.63x10^{5}$	$2.53x10^{6}$	$3.70x10^{6}$	71.99*	29.11
Candida albicans	24	Total	Control						
			Test	$2.37x10^{4}$	$7.86x10^{3}$	$1.74x10^{4}$	$4.05x10^{4}$		
		Viable	Control						
			Test	$3.21x10^{3}$	$7.51x10^{2}$	$2.30x10^{3}$	$4.72x10^{3}$	14.02	2.734
	48	Total	Control						
			Test	$7.65x10^{4}$	$2.72x10^{4}$	$3.87x10^{4}$	$1.13x10^{5}$		
		Viable	Control						
			Test	$3.02x10^{4}$	$1.04x10^{4}$	$1.62x10^{4}$	$4.36x10^{4}$	45.37	23.73
	72	Total	Control						
			Test	$2.35x10^{5}$	$7.75x10^{4}$	$1.26x10^{5}$	$3.17x10^{5}$		
		Viable	Control						
			Test	$9.12x10^{4}$	$1.90x10^{4}$	$6.54x10^{4}$	$1.14x10^{5}$	43.84	20.06
